# A Comparative Analysis of the Effects of Misaligning Different Trifocal Intraocular Lenses

**DOI:** 10.3390/jcm14010187

**Published:** 2024-12-31

**Authors:** Weijia Yan, Gerd U. Auffarth, Ramin Khoramnia, Grzegorz Łabuz

**Affiliations:** 1 The David J Apple Center for Vision Research, Department of Ophthalmology, Heidelberg University Eye Clinic, University Hospital Heidelberg, Im Neuenheimer Feld 400, 69120 Heidelberg, Germany; weijiay@zju.edu.cn (W.Y.); ramin.khoramnia@med.uni-heidelberg.de (R.K.); grzegorz.labuz@med.uni-heidelberg.de (G.Ł.); 2 Eye Center, The Second Affiliated Hospital, School of Medicine, Zhejiang University, Hangzhou 310009, China

**Keywords:** trifocal IOLs, modulation transfer function, optical quality, decentration, tilt

## Abstract

**Objectives:** This laboratory study aims to assess the effects of misaligning different trifocal intraocular lenses (IOLs) under varying spectral and corneal spherical aberration (SA) conditions. **Methods:** With an IOL metrology device under monochromatic and polychromatic conditions, the following models were studied: AT ELANA 841P, AT LISA Tri 839MP, FineVision HP POD F, Acrysof IQ PanOptix, and Tecnis Synergy ZFR00V. The SA was simulated using an aberration-free and average-SA cornea. The modulation transfer function (MTF) was measured at different pupil sizes for the on- and off-axis lens positions. **Results:** The IOLs exhibited varying responses to decentration up to 1 mm, showing the lowest impact in polychromatic light. The least affected was AT ELANA, with an MTF loss of 15.7% to 28.4% at 50 lp/mm across the studied conditions. It was followed by PanOptix and FineVision, with the MTF loss ranging from 19.1% to 36.0% and from 21.2% to 46.6%. AT LISA showed a more substantial reduction, i.e., 41.2% to 64.8%, but it was still lower than that of Synergy (51.1% to 78.8%). When decentration was induced at a 4.5 mm distance, its effect was more evident in conditions that were closer to each IOL’s SA correction. A tilt of 5° had a lesser impact than 1 mm decentration, with the effect being more severe at 4.5 mm. **Conclusions:** The off-axis position affects the optical quality of trifocal IOLs. Low- rather than high-SA-correcting trifocals perform better under misalignment. In polychromatic light, the impact of misalignment is less evident, suggesting a potential mitigating effect of chromatic aberration.

## 1. Introduction

Cataract surgery has evolved beyond its original role of removing the cataractous lens, and, since 1949, replacing it with a clear intraocular lens (IOL) is now a procedure that encompasses lens implantation within a comprehensive surgical approach that rectifies refractive errors and presbyopia [1]. The transformation reflects the growing emphasis on optimizing visual performance across all distances and satisfying the patient’s desire for a reduced reliance on spectacles. As a result, new designs of multifocal IOLs have been developed [2]. Achieving the precise alignment of multifocal IOLs is fundamental in producing optimal visual performance [3]. However, the tilt and decentration of IOLs frequently give rise to a decline in visual quality after cataract surgery [4]. These issues are influenced by various factors, with the patient’s ocular condition playing a central role in determining IOL positioning. Research indicates a significant relationship between IOL tilt and axial length (AL), with tilt decreasing by 0.228° for every 1 mm increase in AL [5]. Additionally, zonular instability is a critical factor, contributing not only to IOL dislocation but also to increased tilt. Surgical techniques such as anterior capsule polishing and the use of capsular tension rings are essential in minimizing IOL tilt and decentration, thus promoting long-term IOL stability and optimal visual outcomes [6]. Although studies have shown that tilts of up to 2–3° and decentration of 0.2–0.3 mm are commonly observed and generally have minimal clinical impact for most IOL designs, larger deviations in tilt and decentration can significantly degrade optical performance, ultimately affecting patient satisfaction [3,4].

Numerous clinical investigations on pseudophakic eyes have used diverse measurement methodologies to estimate the decentration and tilt of IOLs after their implantation [7,8]. Nonetheless, these studies often have limitations, including small sample sizes, challenges in controlling for physiological variables, and a need for simultaneous consideration of the combined effects of tilt and decentration [3]. For instance, while Purkinje reflections are commonly used for IOL alignment, their reliability in clinical settings can be limited by variations in pupil size and lighting conditions [9]. Additionally, the use of the corneal and pupillary centers as alignment references can be confounded by factors such as irregular astigmatism or ocular eccentricities [10]. Moreover, the position of the capsular bag plays a crucial role in IOL centration, with factors like zonular integrity and capsular bag contraction or dilation significantly influencing the final IOL position [11]. There have also been in vitro investigations into the effects of IOL misalignment on imaging quality [12,13,14]. A study by Lawu et al. assessed a selection of six aspheric IOLs, their optical characteristics, and degradation resulting from IOL decentration and tilt [12]. They demonstrated that the aspherical IOL’s susceptibility to misalignment varies depending on the nature of aberration correction. Pérez-Gracia et al. observed that while IOL tilt generally reduces optical performance, this effect is relatively small compared to some IOL design’s sensitivity to decentration [13]. The impact of decentration or tilt does not exhibit consistency across the spectrum of differing multifocal IOL designs [14,15]. Therefore, it is important to assess each IOL’s susceptibility to misalignment, so that the clinician can select the most suitable lens for each patient.

We comprehensively analyzed the visual performances of five aspheric trifocal IOLs with different amounts of spherical aberration (SA) correction. Furthermore, the impact of the decentration and tilt of the IOLs on optical quality was evaluated across scenarios involving monochromatic and polychromatic conditions. This assessment encompasses an aberration-neutral corneal model and a model emulating the average SA levels observed in population studies.

## 2. Materials and Methodology

### 2.1. Intraocular Lenses

Two +20 D lens samples of five trifocal IOLs were evaluated: AT ELANA 841P and AT LISA Tri 839MP (both from Carl Zeiss Meditec AG, Berlin, Germany), FineVision HP POD F (PhysIOL, Liege, Belgium), Acrysof IQ PanOptix (Alcon Laboratories, Fort Worth, TX, USA), and Tecnis Synergy ZFR00V (Johnson & Johnson Surgical Vision, Santa Ana, CA, USA).

The AT ELANA 841P is made of a hydrophobic acrylic heparin-coated material with a diffractive–refractive design and aberration-neutral optics. The lens has a refractive index of 1.49 and an Abbe number of 51 and features an aberration-neutral asphericity. By contrast, the AT LISA Tri 839MP corrects −0.18 μm of corneal SA and has a hydrophilic acrylic platform (refractive index = 1.46 and Abbe number = 56.5) and a hydrophobic surface. Note that the higher the Abbe number, the lower the chromatic dispersion of the IOL material. Both lenses have a trifocal design, with intermediate- and near-focus points at 1.66 D and 3.33 D, respectively.

FineVision HP POD F is manufactured from (26%) hydrophilic acrylic material with a 1.46 refractive index and an Abbe number of 58. The FineVision is a full-diffractive apodized IOL, with the optic having a combination of two diffractive profiles to create three distinct foci at far, intermediate (1.75 D), and near (3.50 D) points. The base lens of the FineVision has an aspheric profile correcting −0.11 μm of (positive) SA of the cornea.

The Acrysof IQ PanOptix is a hydrophobic IOL made of AcrySof material with a 1.55 refractive index and a 37 Abbe number. The PanOptix features a refractive curvature on the anterior surface, while the posterior surface incorporates a diffractive pattern designed to split light into distinct focal points for near-, intermediate-, and far-focus points. The intermediate focus is detected at 2.17 D and the near focus is detected at 3.25 D, providing asphericity of −0.10 μm.

The Tecnis Synergy ZFR00V is a hydrophobic acrylic IOL with a 1.47 refractive index and a 55 Abbe number. The lens corrects 0.27 μm of SA—the average level found in the cataract population [15]. No indication of add powers can be found on lens packaging or instruction for use.

### 2.2. Image Quality Assessment

The optical characteristics of the IOLs were evaluated in adherence with the ISO 11979 standard, using the OptiSpheric IOL PRO2, a metrology apparatus developed by Trioptics GmbH (Wedel, Germany) [16]. This instrument offers a comprehensive analysis of optical parameters, including the effective focal length within a precision of ±0.3% and the modulation transfer function (MTF) with an accuracy of ±2% [17]. The OptiSpheric IOL PRO2 consists of a light source, a test object, a collimator, a modeled eye, a microscope, and a charge-coupled device (CCD) camera. The experimental protocol involves illuminating a test pattern through the collimator and capturing the resultant image projected by an IOL onto the CCD camera. Using a corneal lens with +0.27 µm of SA and polychromatic (Condition 1) and 546 nm (Condition 2) light, as well as with the aberration-neutral cornea model and 546 nm light (Condition 3), each IOL’s optical quality was quantified using the modulation transfer function (MTF). For the evaluation of the MTF, a frequency of 50-line pairs per millimeter (lp/mm) was selected as a reference criterion. The MTF was presented graphically up to 100 lp/mm, and aperture sizes of 3 to 4.5 mm were used to study IOLs’ pupil effects.

The IOL’s tolerance to misalignment was tested by inducing up to 1 mm of decentration in a vertical direction (in 0.1 mm steps); thus, only the sagittal (vertical) component of the crosshair target was analyzed. Furthermore, the 5° tilt was simulated using a dedicated insert provided by Trioptics GmbH. Following IOL tilt, both MTF meridians were analyzed and averaged for comparison.

## 3. Results

### 3.1. Far-Focus MTF Analysis

**Figure 1:**
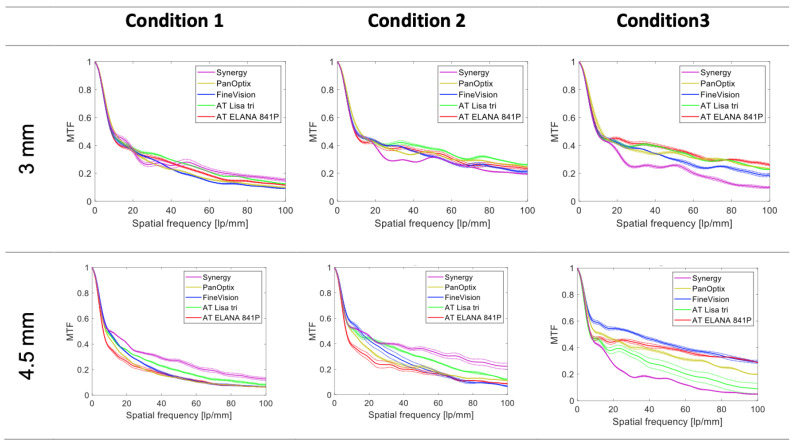
MTF levels of the trifocal IOLs at the far-focus point. The dotted lines show the values of each lens separately; the solid lines refer to the average of two samples. Condition 1 = polychromatic light and SA = +0.27 µm; Condition 2 = 546 nm light and SA = +0.27 µm; Condition 3 = 546 nm light and aberration-neutral cornea model.

Figure 1 shows the average MTF curves of the studied IOLs at 3 and 4.5 mm and the three conditions. At photopic pupil sizes (3 mm), the trifocal IOLs demonstrated comparable performance in polychromatic light. In Condition 2, Synergy’s optical quality slightly declined in contrast to AT LISA’S and AT ELANA’s improvements. The introduction (in Condition 3) of the aberration-neutral cornea further deteriorated Synergy’s performance, with the AT ELANA producing the highest MTF levels. Still, the differences between the other models were minimal.

The MTF results obtained at the scotopic pupil size (4.5 mm) demonstrated that a highly aberrated condition favored the far-point performance of the Synergy in contrast to the AT ELANA, the PanOptix and the FineVision. In Condition 2, the comparison between the models yielded a similar conclusion, but the gap between them widened. However, it was reversed with the aberration-neutral corneal model, which improved FineVision’s and AT ELANA’s MTF values and worsened Synergy’s MTF values.

The far-focus MTF obtained for the trifocal IOLs at 3 and 4.5 mm served as a basis for comparing the effect of misalignment on their optical performance.

### 3.2. IOL Decentration

**Figure 2:**
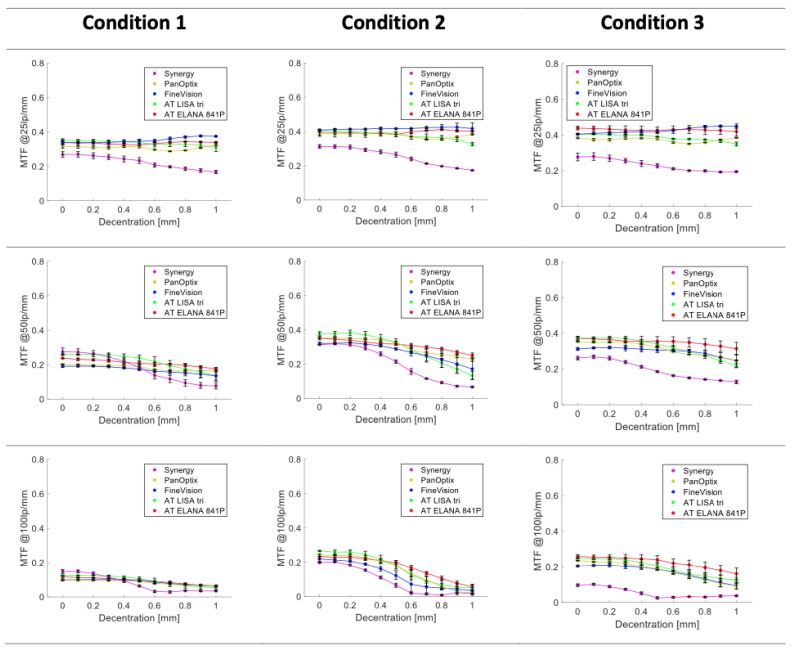
Average values of far-focus MTFs at 25, 50, and 100 lp/mm measured with trifocal IOLs under lens decentration for a 3 mm pupil. The error bars indicate the differences between the two samples. Condition 1 = polychromatic light and SA = +0.27 µm; Condition 2 = 546 nm light and SA = +0.27 µm; Condition 3 = 546 nm light and aberration-neutral cornea model.

**Figure 3:**
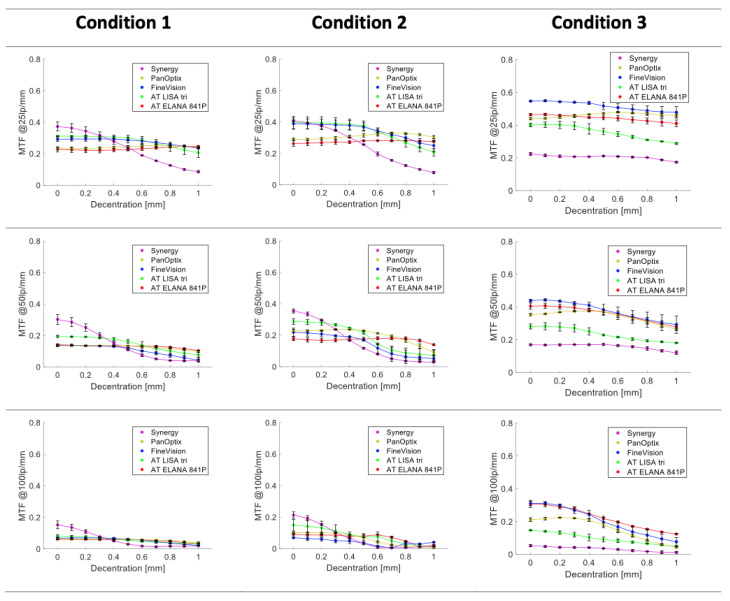
Average values of far-focus MTFs at 25, 50, and 100 lp/mm measured with trifocal IOLs under lens decentration for a 4.5 mm pupil. The error bars indicate the difference between the two samples. Condition 1 = polychromatic light and SA = +0.27 µm; Condition 2 = 546 nm light and SA = +0.27 µm; Condition 3 = 546 nm light and aberration-neutral cornea model.

The IOLs showed varying levels of tolerance to decentration. Figure 2 and Figure 3 present a discrete MTF value at 25, 50, and 100 lp/mm as a function of decentration (up to 1 mm) for the best far-focus point determined at the 3 and 4.5 mm apertures.

At 3 mm, the AT ELANA demonstrated a minimal change in its 25 lp/mm performance and more pronounced deterioration at higher spatial frequencies. At 25 lp/mm, the optical-quality change between on- and 1 mm off-axis positions ranged from −4.3% (Condition 3) to +2.6% (minimal improvement; Condition 2); for the 50 lp/mm, it ranged from −28.4% (Condition 2) to −15.7% (Condition 3); and for 100 lp/mm, it ranged from −75.7% (Condition 2) to −37.8% (Condition 3). The AT LISA’s imaging quality was more affected by decentration with a 50 lp/mm loss of −47.8% (Condition 1), −64.8% (Condition 2), and −41.2% (Condition 3). Unlike the AT ELANA, the AT LISA demonstrated a more pronounced loss of low-frequency MTF at 1 mm decentration, ranging from −19.4% to −12.4%. The 100 lp/mm MTF was more substantially affected in Condition 2 (−81.3%) than in Conditions 1 (−51.7%) and 3 (−49.9%). The Synergy demonstrated a steady decline in the MTF level, noticeable at all spatial frequencies and conditions. The maximum optical-quality deterioration was in Condition 2, which yielded −44.4% (25 lp/mm), −78.6% (50 lp/mm), and −91.0% (100 lp/mm). Synergy’s image quality was the least affected in Condition 3, with −29.7% (25 lp/mm), −51.1% (50 lp/mm), and −61.3% (100 lp/mm). Still, Synergy’s MTF loss was not comparable to any other model. The FineVision revealed an improvement range of +2.3% (Condition 2) to +11% (Condition 1) at 25 lp/mm, as well as deterioration from −46.6% (Condition 2) to −21.2 (Condition 3) at 50 lp/mm and from −84.4% (Condition 2) to −37.9% (Condition 1) at 100 lp/mm. For the PanOptix, the 25 lp/mm values ranged from −1.9% (Condition 2) to +2.5% (Condition 1), from −36.0% (Condition 2) to −19.1% (Condition 1) for 50 lp/mm, and from −98.1% (Condition 2) to −34.3% (Condition 1).

The trend at 4.5 mm was similar to that observed at 3 mm. The Synergy was the most susceptible to decentration at all spatial frequencies, while the AT ELANA was the most robust. Only in Condition 3, and at 100 lp/mm, can an evident MTF decline in the AT ELANA be observed with a −59.7% loss. Figure 3 compares all models under the three conditions and the discrete frequency levels.

### 3.3. IOL Tilt

Table 1, Table 2 and Table 3 report on the tilt simulations for each condition measured at 3 mm and 4.5 mm. Overall, a tilt of 5° appears to affect the MTF to a lesser extent than 1 mm decentration.

In Condition 1, the MTF value at 100 lp/mm was comparable before and after 5° tilt for the AT ELANA and the PanOptix. A −0.03 MTF loss was observed in the FineVision and the AT LISA, followed by the Synergy (−0.04). In Condition 2, the AT ELANA produced the smallest tilt effect (−0.04) compared to the AT LISA and the Synergy (both −0.08). The FineVision and the PanOptix yielded reductions of −0.05. In Condition 3, the MTF loss at 100 lp/mm for the PanOptix was −0.05, FineVision’s was −0.07, Synergy’s was −0.04, AT ELANA’s and AT LISA’S was −0.09.

At 4.5 mm, the AT ELANA and the PanOptix demonstrated an MTF loss of −0.01 at 100 lp/mm in Condition 1. A minimal change was observed in the FineVision (−0.02), followed by the AT LISA (−0.03), and the Synergy (−0.04). In Condition 2, the AT ELANA, the PanOptix, the AT LISA, and the FineVision were virtually not affected by 5° tilt, but the Synergy exhibited a noticeable loss of −0.09. In Condition 3, the FineVision produced the largest loss of the MTF value at 100 lp/mm, i.e., −0.22. A loss of −0.22 was found for the AT ELANA and −0.14 for the PanOptix. By contrast, the AT LISA and the Synergy were minimally affected (both −0.02).

## 4. Discussion

In the present study, five contemporary trifocal IOL technologies were compared, providing an objective measure of optical quality and the effects of IOL decentration and tilt.

Previous studies highlighted the adverse effects of significant decentration and tilt on postoperative optical performance and patient satisfaction. For instance, Guyton et al. reported that visual quality can be compromised when decentration exceeds 1 mm or tilt greater than 5° [18]. Similarly, Hayashi et al. found that the distant mean LogMAR visual acuity (VA) did not reach 0.20 when decentration equaled or exceeded 0.9 mm [19]. Our study corroborates these findings, showing a decline in optical quality for all five trifocal IOLs when decentration reached 1 mm. Although decentration of the IOLs reaching 1 mm is rare, a study by Crnej et al. involving 15 patients (30 eyes assessed) reported that one eye in the analysis of the plate-haptic configuration exhibited decentration close to 1 mm. In their subsequent comparison between one-piece and three-piece IOLs, decentration exceeding 1 mm was observed in 2 eyes out of the 30 assessed [8]. Notably, the Synergy IOL exhibited a greater reduction in visual quality across a range of spatial frequencies and under varying conditions compared to the others. However, not all studies agree on the impact of decentration. Negishi et al. conducted research on both monofocal and multifocal IOLs and concluded that deviations of up to 1.0 mm did not significantly compromise VA [20]. This variability in findings may arise from the different methods used to assess the impacts of tilt and decentration, as well as variations in IOL technology. Still, the association of misalignment with the degree of asphericity has been documented in clinical investigation [21].

Baumeister et al. analyzed decentration and tilt in spherical and aspherical IOLs [7]. They showed that the mean optic tilt was 2.89° ± 1.46 (SD) for the spherical IOL and 2.85° ± 1.36° for the aspheric IOL. The mean optic decentrations were 0.19 ± 0.12 mm and 0.27 ± 0.16 mm, respectively, three to four months after implantation. Crnej et al. presented their clinical findings from the misalignment of plate-haptic IOLs versus open-loop one- and three-piece AcrySof lenses [8]. At the three-month postoperative examination, the tilt measurements varied between −1.9° ± 1.4° and 2.9° ± 0.9° for the plate-haptic IOL group, between 2.6° ± 4.1° and 5.3° ± 2.4° for the three-piece IOL group, and between 1.9° ± 0.3° and 2.2° ± 7.2° for the one-piece IOL group. As for decentration, the range spanned from 0.07 ± 0.28 mm to 0.34 ± 0.15 mm after the implantation of plate-haptic IOLs, from −0.08 ± 0.57 mm to 0.32 ± 0.48 mm with three-piece IOLs, and from 0.08 ± 0.30 mm to 0.19 ± 0.20 mm with one-piece IOLs. Comparable outcomes were reported by Gu et al., who measured the stability of a hydrophilic acrylic IOL and reported the mean tilt and decentration of the IOL to be 4.75° ± 1.66° and 0.21 ± 0.02 mm postoperatively [22]. We can note that the observations in these empirical studies align with the range of results that we observed in our laboratory simulations, thus establishing a connection between our simulated scenarios and these clinical results.

Furthermore, our investigations into visual image analyses reveal a clear and notable correlation between the extent of aberration correction and the occurrence of decentration or tilt. This significant relationship is visually depicted in Figure 2 and Figure 3. Specifically, the Synergy model stands out for its heightened vulnerability to decentration across the entire range of spatial frequencies. This susceptibility can be attributed to Synergy’s ability to provide a substantial degree of negative spherical aberration correction, a feature distinguishing it from other presbyopia-correcting IOLs. These findings align with the research conducted by Pieh et al. [23] and that of Lawu et al. [12], both of which support the view that in IOL designs that incorporate a higher degree of negative spherical aberration, the optical degradation is more evident when the IOL is misaligned.

**Figure 4:**
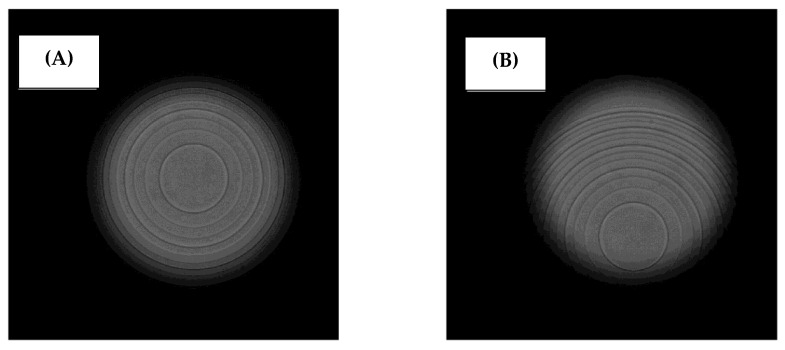
Images of the PanOptix lens with an on-axis position (**A**) and after 1 mm decentration (**B**).

The AT ELANA exhibited good tolerance to IOL decentration, which may stem from its aberration-neutral design. A low-aspheric design has proven more robust against tilt and decentration in numerous studies [24,25,26]. Interestingly, this lens, the FineVision, and the PanOptix demonstrated a slight MTF improvement beyond a 0.5 mm decentration. This rather unexpected result can be explained for the PanOptix by noting that a smaller portion of the light passes through the diffractive element because more than half of its structure falls beyond the pupil area (Figure 4).

As a result, rays that miss the diffraction grating are not split, which favors the far-focus point by increasing its partition of light. A similar outcome has been reported for a supplementary trifocal IOL featuring an incomplete diffractive design [27]. The improvement of the optical quality at the far-focus point came, however, at the expense of the intermediate- and near-focus points since a smaller portion of light contributes to these points when the light travels through the lens-optic periphery. For the FineVision, its apodization increases the proportion of light reaching the far-focus point at larger apertures and may contribute to a similar effect. For the AT ELANA, a slight improvement of low spatial frequencies was observed. Still, higher spatial frequencies were compromised (to a different extent) for all models and in all conditions. Therefore, to optimize the performance of a trifocal IOL, it is essential to ensure proper lens centration.

We demonstrated that the impact of a 5° tilt on the optical quality depends on the lens model, the aperture, and the experimental condition. At 3 mm and in Condition 1, the largest MTF difference at 50 lp/mm was −0.04 found in the FineVision model, which was less or comparable to the MTF loss reported in the study on IOLs with an extended depth of focus [24]. But Condition 1 was the least affected by tilt, perhaps because of the presence of chromatic aberration, which can introduce wavelength-related blurring or smearing, which may smooth out or partially mask the detrimental effects caused by IOL tilt, mitigating its effects. In Conditions 2 and 3, the largest MTF loss was MTF = −0.08 to −0.09, observed at 100 lp/mm, with 25 lp/mm being mostly unaffected, indicating that IOL tilt affects primarily higher spatial frequencies. The effects of tilt were exacerbated at 4.5 mm aperture in Condition 3, which may be related to a stronger effect of oblique aberrations. According to the aberration theory, the coma aberration is proportional to the aperture cubed, and astigmatism is the aperture squared. The pupil dependency of the higher order aberrations and the lack of chromatic (Condition 1) and spherical (Conditions 1 and 2) aberration may explain a stronger effect of IOL tilt observed at all spatial frequencies in Condition 3 (4.5 mm), particularly for the AT ELANA. Still, the effects were marginal in polychromatic light and with spherical aberration, which may have a compensatory impact on these oblique aberrations. Interestingly, after tilting in Conditions 1 and 2, a slight improvement in performance was demonstrated in the AT ELANA. We attribute this observation to the measurement error and reproducibility issue at higher apertures.

## 5. Conclusions

We demonstrated the varying responses of trifocal IOLs to misalignment, such as decentration and tilt, which may more closely represent real-world scenarios. While all lenses produced good and comparable performance after on-axis placement, one can distinguish between their optical designs. In particular, the Synergy shows a substantial reduction in optical quality with increasing misalignment. This is an outcome that needs to be considered when there is an expectation of postoperative lens misalignment. By contrast, the AT ELANA’s aberration-neutral design appears more forgiving in cases where optimal centration cannot be guaranteed. Further studies are needed to elaborate on the impact of IOL misalignment on visual quality and its role in exacerbating the perception of photic phenomena in patients with trifocal lenses. The study underscores the significance of standardized measurement techniques for assessing IOL tilt and decentration, which would enhance outcome comparability and improve surgical practices. It further highlights that IOL misalignments, including tilt and decentration, can adversely affect the optical quality, and thus visual function, particularly with aspheric and multifocal IOLs, thereby emphasizing the critical need for precise IOL placement. However, the impact of decentration and tilt has only been assessed at the far-focus point. Further studies could extend this analysis by measuring MTFs at intermediate- and near-focus points to better understand the full performance profile of trifocal IOLs.

## Figures and Tables

**Table 1:** Comparison of the MTF values taken under 5° tilt vs. no tilt at 3 and 4.5 mm in Condition 1 (polychromatic light and SA = +0.27 µm). Negative and positive values represent MTF loss and improvement, respectively.

	3 mm			4.5 mm		
	Δ (25 lp/mm)	Δ (50 lp/mm)	Δ (100 lp/mm)	Δ (25 lp/mm)	Δ (50 lp/mm)	Δ (100 lp/mm)
**AT ELANA**	0.01	−0.01	−0.02	0.01	−0.01	−0.01
**AT LISA**	−0.02	−0.03	−0.03	−0.04	−0.05	−0.03
**FineVision**	−0.03	−0.04	−0.03	−0.01	−0.03	−0.02
**PanOptix**	0.00	−0.01	−0.01	−0.01	−0.01	−0.01
**Synergy**	−0.01	−0.03	−0.04	−0.02	−0.04	−0.04

**Table 2:** Comparison of the MTF values taken under 5° tilt vs. no tilt at 3 and 4.5 mm in Condition 2 (546 nm light and SA = +0.27 µm). Negative and positive values represent MTF loss and improvement, respectively.

	3 mm			4.5 mm		
	Δ (25 lp/mm)	Δ (50 lp/mm)	Δ (100 lp/mm)	Δ (25 lp/mm)	Δ (50 lp/mm)	Δ (100 lp/mm)
**AT ELANA**	0.02	−0.02	−0.04	0.04	0.02	0.00
**AT LISA**	−0.02	−0.06	−0.08	−0.07	−0.05	−0.01
**FineVision**	0.01	−0.03	−0.05	−0.03	0.01	0.01
**PanOptix**	−0.01	−0.03	−0.05	0.00	0.00	−0.02
**Synergy**	−0.01	−0.03	−0.08	−0.04	−0.07	−0.09

**Table 3:** Comparison of the MTF values taken under 5° tilt vs. no tilt at 3 and 4.5 mm in Condition 3 (546 nm light and aberration-neutral cornea model). Negative and positive values represent MTF loss and improvement, respectively.

	3 mm			4.5 mm		
	Δ (25 lp/mm)	Δ (50 lp/mm)	Δ (100 lp/mm)	Δ (25 lp/mm)	Δ (50 lp/mm)	Δ (100 lp/mm)
**AT ELANA**	−0.02	−0.05	−0.09	−0.13	−0.22	−0.22
**AT LISA**	−0.01	−0.06	−0.09	−0.01	−0.05	−0.02
**FineVision**	0.00	−0.03	−0.07	−0.1	−0.24	−0.24
**PanOptix**	0.00	−0.02	−0.05	−0.1	−0.12	−0.14
**Synergy**	−0.02	−0.03	−0.04	0.02	−0.01	−0.02

## Data Availability

Data are contained within the article.
